# Jewish/non-Jewish encounters in corridors and staircases: narrowing down everyday life in liminal spaces

**DOI:** 10.1080/1462169X.2024.2319414

**Published:** 2024-02-28

**Authors:** Maja Hultman, Susanne Korbel

**Affiliations:** a Centre for European Research/Department of Historical Studies, University of Gothenburg, Gothenburg, Sweden; b Center for Jewish Studies, University of Graz, Graz, Austria

**Keywords:** Urban space, liminality, shared spaces, residences, Vienna, Stockholm

## Abstract

Contrary to historiographical narratives about Jewish seclusion in European cities at the turn of the twentieth century, we argue that Jews and non-Jews mingled and developed relationships on a daily basis in residential and everyday spaces. We develop the concept of liminal topographies to show how the transient, in-between material structures of staircases and corridors in two such disparate case studies as Stockholm and Vienna facilitated Jewish/non-Jewish relational arenas for the broader masses. Combining digital mapping, visual analysis and text analysis, this approach expands the field of Jewish spatiality by underlining the link between absolute spaces and relational processes.

## Introduction^1^

In the years since I was a child, the loggias have changed less than other places. This is not the only reason they stay with me. It is much more on account of the solace that lies in their uninhabitability for one who himself no longer has a proper abode. They mark the outer limit of the Berliner’s lodging. Berlin – the city god itself – begins in them.^2^

Walter Benjamin, from ‘Loggias’ in *Berlin Childhood around 1900*

Looking back at his childhood in Berlin at the end of the nineteenth century, Walter Benjamin positioned himself on a loggia, a room with one or more open sides towards the courtyard. As a refugee from the Nazi regime in the 1930s, Benjamin imagined the architectural structure as a ‘mausoleum’ of his bourgeois, shattered past. Suspended in the air between his family’s home and the courtyard below, the loggia is, however, also introduced as a space of connection, bridging Benjamin-as-a-child and the larger urban world. As the writer noted in the quoted section above, the loggia is a material structure located in-between the private and the public. It marks the absolute end of a person’s apartment yet is involved in the surrounding city life. In other words, the loggia is a liminal space.

While Benjamin distinctly associated the loggia, and his memories of it, with modern Berlin, similar spatial nooks, architectural punctuations, and material openings define all urban milieus. As examples of private or semi-private spheres, streets and alleyways in the public sphere, as well as balconies, thresholds, and corridors, have been categorized and analysed as liminal structures enabling – even provoking – encounters among city dwellers in the modern city.^3^ These studies are part of the so-called ‘spatial turn’ that has emerged in the last decades as an unavoidable set of methods and theoretical reflections in cultural studies and the Humanities in general, and in Jewish studies in particular. Approaches to spatiality in Jewish studies have outraged into multifarious directions, spanning Judaic studies to cultural studies and Digital Humanities. Yet, the impact of spatial analysis has sometimes remained vague, even more so in examples investigating liminal spaces. What benefits does the spatial lens offer to conventional historiography? How can a liminal space – as an analytical and largely abstract concept – be tangibly narrowed down for historical studies? Or to put it even more provocatively: Is a liminal space more than a sheer label to impose on Jewish studies?

In this article, we claim that an in-depth spatial investigation that targets multiple layers offers new, unexpected findings that complement, enhance, and sometimes even revert conventional historiographies’ interpretation of Jewish/non-Jewish everyday relations. Prompted by results from digital mapping tools, we perform a close visual and textual analysis of liminal spaces in the historic pasts of two very different European cities, inhabited by heterogeneous Jewish populations: Stockholm and Vienna. We show that diverse spatial methods can be productively combined under the concept of ‘liminal topography’ to demonstrate that Jewish life in the capitals of Sweden and Austria carried comparable relational opportunities with each city’s non-Jewish population. In so doing, our analysis presents how the bridging of the two poles of relational and digital approaches within the field of spatial Jewish history offers a powerful transnational comparison of European Jewish urban life at the turn of the twentieth century. The article is divided into four sections: First, we present digital mappings of Jewish settlements in Stockholm and Vienna, and show how these findings contradict earlier historiography, inviting us to ask new questions about urban interactions among Jews and non-Jews at the end of the nineteenth century and the beginning of the twentieth century. As a second step, we address considerations of and approaches to spaces and human interactions within them, arguing for the necessity of a stronger introduction of liminal spaces in Jewish studies. Third, we examine liminal topographies, giving the examples of staircase and corridor settings in Stockholm and Vienna, to show how a liminal approach can unearth new dimensions of the European Jewish experience of the modern metropolis. Finally, conclusions are drawn on how liminal topographies – the tangibly narrowing down of liminal spaces – provide a pathway for relational trajectories in future investigations of Jewish/non-Jewish everyday encounters, particularly in the six or so decades before the rise of National Socialism.

## Digital mapping of Jewish populations in Stockholm and Vienna

It might seem daring, perhaps even irrelevant, to compare and contrast Jewish life in Stockholm and Vienna at the turn of the twentieth century. It is not only a question of the latter being known as *the* European capital of modernity, while the Swedish capital just entered the social and industrial processes associated with the modern era. The Jewish populations in the two capitals also had different historical backgrounds. Having only been allowed to live as practicing Jews in Sweden since 1775, the Jewish population in Stockholm was numerically small, numbering some 2,500 among Stockholm’s 300,000 individuals in 1900. The Jewish community of Ashkenazi German and Eastern European-descendant individuals was emancipated in 1870 and largely aligned to Reform Judaism and acculturation processes. As a difference to the relatively short Jewish presence in Sweden, Jews have lived in Vienna since the early Middle Ages. Between persecution and expulsion, toleration and mutual exchange, Jewish life was able to grow in all areas of society after the Tolerance Patents of Joseph II. With the official foundation of the second Viennese Jewish community in 1852, and the full emancipation in 1867, Jews were integral to the Viennese society until National Socialism’s seizure of power.

While both cities were affected by the European continent’s westward migration during the nineteenth and twentieth centuries, which pulled some two million Jews from the Russian Empire and the Eastern parts of the Habsburg territories through Central Europe towards the dream of America, the polarised scales of arriving migrants created vastly different socio-economic and spatial settings for Jewish inhabitants and migrants. Vienna was a port of call for numerous migrants and thus experienced a veritable explosion in population. The three-times increased population between 1890 and 1930 turned the capital of the Habsburg Empire into a city of two million. Every second Viennese was not born in the city they lived in,^4^ and institutions were soon established to facilitate Jewish (trans)migration and community life. The demographic changes also made Vienna a home to the third largest European Jewish community, comprising approximately 147,000 individuals in 1900,^5^ and 175,000 in 1910.^6^ In contrast, the route across the Nordic countries was not a favoured journey among migrants. Indeed, the last 50 years of the nineteenth century even saw a fifth of the Swedish population leave for the United States due to starvation, agricultural reforms, and a comparably slow development of industrialisation. Despite this, some 3,000–4,000 Eastern European Jews arrived in Sweden between the 1860s and 1917,^7^ prompting the already established Jewry to set up philanthropic institutions to improve the lives of their poorer brethren and prevent expected antisemitic backlash. The incomparability between population scales and historical temporality for Jewish life in Stockholm and Vienna created two very different locales, which, as we will see later, nonetheless both offered numerous opportunities for Jewish and non-Jewish encounters in everyday life. By choosing these two case studies, we get access to transnational similarities of Jewish/non-Jewish quotidian togetherness across a diverse spectrum of living realities within the modern European Jewish experience.

Interestingly, despite the cities’ different urban histories, conventional historiographies of Jewish representations in the urban landscapes of Stockholm and Vienna adhere to similar trajectories. In the Swedish example, historians have until the last couple of years regarded the Jewish population in Stockholm as socially, economically, and religiously dichotomized, dividing them between German-descendant, integrated, and reformed Jews, and Eastern European, poor, and mostly orthodox immigrants. Anna Besserman argued in 1984 that ‘the only existent contact space was between the needy and the philanthropists.’^8^ Despite no dedicated spatial study, the dichotomized image between established Jews and Eastern European Jews has, furthermore, been inscribed into urban space by scholars and public educators alike, creating a narrative of ‘northern Jews’ and ‘southern Jews’.^9^ Within this paradigm, in which the German-descendant ‘northern Jews’ have been placed closer to the cultural and social trajectories of the larger society, an investigation of the everyday relationship between non-Jews and Eastern European Jews has received little scope, creating a sense that it did not even exist.^10^

Similarly, conventional historiography portrays the daily lives of Viennese Jews at the outset of modernity as secluded, mostly determined by non-contact with, or even avoidance of, non-Jews.^11^ This narrative is a result of research in the 1980s that had not yet access to rich sources and in-depth digital and spatial analysis. These no less important early studies conceptualized the social patterns of Vienna’s Jewish population as exclusive and suggested that Jewish/non-Jewish co-residence was close to non-existent at the turn of the twentieth century. For instance, Marsha Rozenblit reached the following conclusion regarding Jewish/non-Jewish everyday relations:

The Vienna Jews lived with other Jews. The creation of Jewish neighborhoods in the city served to separate Jews from gentiles and install more deeply the perception – among Jews and non-Jews alike – that Jews formed a distinct group. Within their neighborhoods, Jews came into contact chiefly with other Jews. Their residential concentration thus hindered them from forming friendships and other intimate relationships with non-Jews.^12^

Later scholarship has attempted to soften the depiction of a distinct and isolated Jewish pattern of everyday life. For example, Ivar Oxaal found that Jews lived next door to non-Jews before the First World War, and Klaus Hödl illuminated multiple fields of Jewish/non-Jewish togetherness in the urban fabric.^13^ Nevertheless, a supposed Jewish inaccessibility to some spaces in the cityscape remains perpetuated by historical scholarship and in literary, cultural, and social studies alike.^14^

In both case studies, the longevity of decades-old historiography has dictated Jewish modern urban life to be in parts segregated from the non-Jewish world. Our adoption of digital mapping tools, however, suggests a different story. Below, we present three maps of Jewish settlement patterns and spatial routines in Stockholm and Vienna respectively. Due to a lack of space, readers interested in discussions of our methods are advised to turn to our previous publications.^15^ By using the analytical and digital approach of HGIS (Historical Geographic Information Systems), we are tapping into the resurfaced field of Jewish digital history^16^ and its increasing number of studies that re-evaluate historical spaces and historiographical presumptions, pushing Jewish history into new premises.^17^ Similarly, we argue that our digital and spatial analysis visualizes a more nuanced picture of Jewish everyday life in two European urban environments at the beginning of the twentieth century: The results from our digital analysis propose recurring, everyday Jewish/non-Jewish encounters in public, private, and also liminal spaces.

**Map 1.**
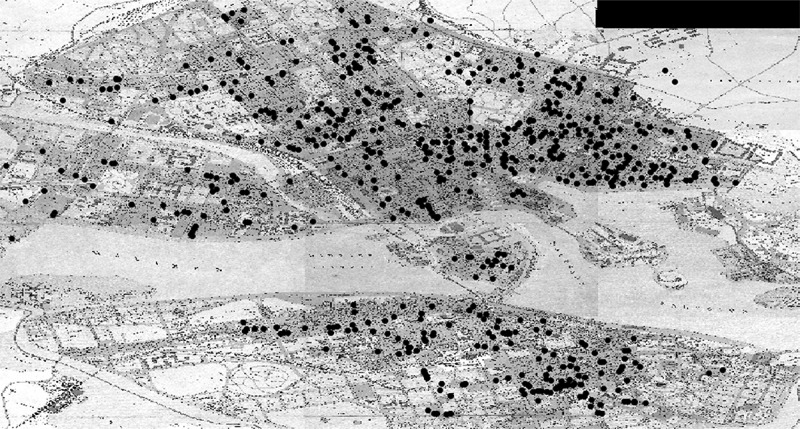
Settlement pattern of Stockholm’s Jewish population in 1909. Map from *Stockholms stadsarkiv*.

As can be seen in Map 1, the Jewish population in Stockholm lived in a fragmentary pattern across the whole city. The map represents the homes of 1,527 mainly male adults of Stockholm’s approximately 2,600 Jews (including women and children) in 1909.^18^ Contrary to previous scholars’ perception of Jewish life in the Swedish capital, there are no clusters or areas with a visibly concentrated Jewish population. Instead, Jews seemingly settled across the whole width and breadth of the city, from the modernised city centre, north of the island of Old Town, to the industrial suburbs and cheap apartment buildings in all cardinal directions. Without the geographical segregation proposed by earlier scholars, Jewish life – whether it was practiced by established or Eastern European Jews, reformed or orthodox Jews, rich or poor individuals – could not have been secluded from the non-Jewish population. Instead, orthodox and/or Eastern European Jews lived and moved among non-Jewish neighbours, sharing not only public spaces in the city, but also – or perhaps even more so – thresholds, corridors, staircases, and courtyards.

**Map 2.**
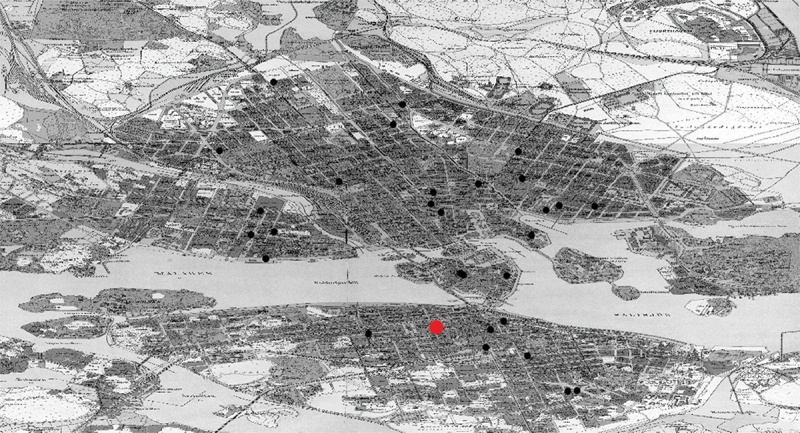
Settlement pattern of Jews petitioning for a *Talmud Torah* in Stockholm, 1925. Map from *Stockholms stadsarkiv*.

Map 2 zooms in on the residential distribution of 33, again, mainly male adults who signed a petition to the Jewish community in Stockholm in 1925 for the establishment of a *Talmud Torah*, a religious afternoon school that was dedicated to traditional rituals and practices. Over four fifths of the individuals who signed the petition were under the age of 55, with a third below the age of 45, which suggests that many of them had children that would attend the orthodox afternoon school. Only a minority of them lived in southern Stockholm, the area prescribed by Swedish historiography to orthodox Jews. Indeed, the distribution of residences that aligned to orthodox practices mirrors the larger Jewish population’s fragmentary pattern of settlement.^19^ When the *Talmud Torah* was established in the orthodox synagogue of *Adass Jisroel* (red dot on Map 2), initially without support from the main community, children walked or took the tram between their homes and the synagogue several times a week to participate in classes. Spatial practices linked to an orthodox lifestyle would have taken most children across vast areas of Stockholm, out from apartment buildings shared by Jewish and non-Jewish residents, through both poorer and richer neighbourhoods, and across thoroughfares and water bodies. Contrary to the historiography described above, Swedish Jewish orthodox life was embedded in the cityscape and included regular intersections with the non-Jewish majority in both public and residential spaces.

**Map 3.**
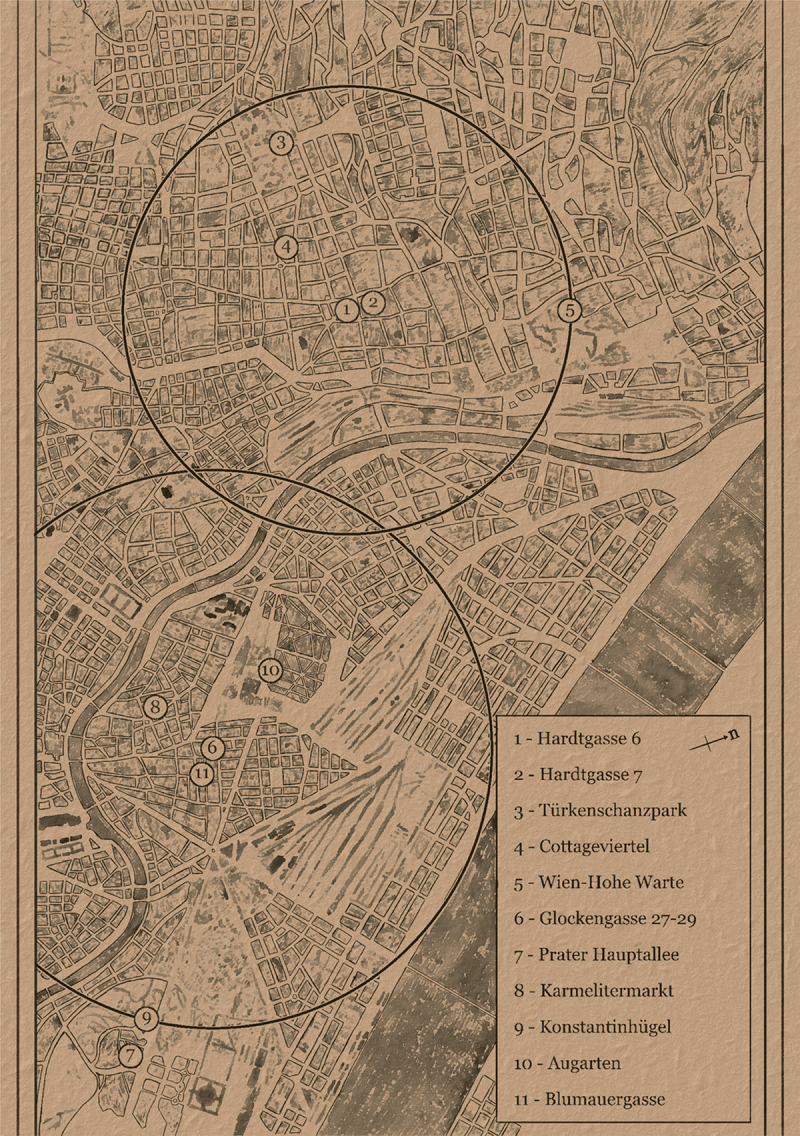
Spatial routines of two Viennese protagonists in the early twentieth century. Open Street Map based, enriched with GIS from the *Wienerkulturgutplan 1912*, Wiener Stadt- und landesarchiv.

Moving on to Vienna’s Jewish population, the investigation of the scholarly perception of the community’s spatial isolation is facilitated through a qualitative approach. Here, a picture similar to Stockholm’s Jewry emerges when looking at contacts, separateness, and transitions between Viennese Jews and other Austrians. Map 3, which is based on the two autobiographies *Glockengasse 29* and *Hardtgasse 6* (presented in more detail below), illustrates Jewish daily routes in the cityscape, indicating which urban areas were part of the protagonists’ everyday life.^20^ The authors lived in two different districts, namely on *Glockengasse* 29 in *Leopoldstadt* and on *Hardtgasse* 6 in *Döbling*. At the turn of the twentieth century, *Leopoldstadt* was renowned as the area of Jewish settlement since the early modern times and housed not only synagogues and other Jewish institutions, but also the northern railway station, which connected the capital to the Habsburg Empire’s eastern provinces, and consequently made *Leopoldstadt* the first port of call for Jewish migrants from Eastern Europe. *Döbling*, on the other hand, was considered as a supposedly less ‘Jewish’ district.

In their autobiographies, both authors describe encounters with people – Jews and non-Jews alike – as they are on the move between various places. Map 3 visualizes these opportunities to encounter Jewish and non-Jewish citizens: the larger circles indicate the walking distance of approximately 30 minutes from each protagonist’s home address. As can be seen, neither *Leopoldstadt* nor *Döbling* facilitated a non-contact spatial experience. Indeed, the analysis prompted by Map 3 interrogates Jewish exclusivity in the allegedly ‘Jewish’ district of *Leopoldstadt* by considering living habits in public space as a mutual Jewish/non-Jewish project.^21^ While many Jews continued to live in the district in the modern era, prompting the nickname ‘Mazzesinsel’,^22^ Jews were by no means the only ethnic group. Counting around 3,000 houses, the district was the most densely populated part of the city for both Jews and non-Jews. People shared sanitary facilities and dormitories in apartment buildings, Jews and non-Jews regularly met in the street, and leisure time was spent side by side in neighbouring parks.^23^

That people encountered one another in the street, as the quantitative illustrations of Stockholm also make evident, is perhaps not surprising, although this aspect is rarely considered in studies of urban becoming. What is striking, however, is that all three maps suggest that private residential areas served as spaces of connection between Jews and non-Jews. To reach an even thicker description of Jewish everyday life in urban contexts, to approach what ‘deep maps’ aim at,^24^ we need to search for Jewish/non-Jewish contacts beyond public spaces. Thus, the digital analysis achieves two things: It prompts us to question earlier paradigms established within the historiographies of Stockholm’s and Vienna’s Jewries and to re-assess the nature of Jewish/non-Jewish relations, and it anchors Jewish/non-Jewish encounters in specific material structures. The spatial macro analysis of the cities invites us to zoom further into the urban composition and ask what kind of sociability these urban topographies of contact facilitated. This is in line with Martina Löw’s request for a new sociology of space that includes wide relational aspects, including determents of contacts, sociability, and relations.^25^ While Löw’s finding is anchored in our contemporary world, it can also be applied to historical questions regarding encounters, intermingling, and co-presence in spaces. As the example of the loggia above suggests, the design of residential spaces can provide in-between, transgressive, and bridging – liminal – material structures. Prompted by our digital work towards this kind of space, we now dive into the spatial approach in Jewish studies to assess what the conceptualization of liminality as a relational space can add to the field.

## Towards liminal spaces in Jewish studies

Following a momentary burst of interest in Marxist theory and the Annales, spatial considerations increasingly came to the forefront of research from the late 1980s onward. Since then, space-based concerns have replaced the time-dominated focus of historiographical and cultural studies,^26^ developing concepts such as place and space, as well as interstices, borders, contact zones, and transitional areas. The results of these perspectives are diverse, even bordering fragmentation,^27^ but common to all of them is an emphasis on social relations or cultural intersections. Henri Lefebvre understands spaces as products of social processes. Spaces become and exist through and in human interactions; they come into being, as Michel de Certeau puts it.^28^ Conversely, spaces also determine the human encounter in them.^29^ Spaces are thus fleeting and temporary: They appear, evolve, and disappear.^30^

Research has long tended to separate place from space – a distinction that has often entailed an opposition of the two concepts.^31^ One understanding defines place as the basis for the production of social space. Lefebvre, for example, states that ‘[e]very space is already in place before the appearance in it of actors.’^32^ De Certeau distinguishes relationally constituted space from place as its (physical) determination of location.^33^ Whereas, for the other understanding, spaces are merely a container that becomes a relational product – a place – by imposing meanings through contacts and relations.^34^ Connecting the dichotomy productively, Yi-Fu Tuan intertwines the two when he argues that social space also becomes a place when ‘we get to know [it] better and endow it with value’.^35^ In other words, Tuan proposes a closer conceptual relationship between physical locations and the meanings attached to, and relations performed in, them.

The spatial turn arrived comparatively late in Jewish Studies,^36^ and despite spatial theory’s promise of a dedicated focus on the interdependency between topography and the relational dimension, it has unfolded in limited directions.^37^ Due to narratives dealing with diaspora and the history of Jewish identity formation, questions about Jewish spaces have long been related to territoriality or fixed on religious aspects. Miriam Rürup and Simone Lässig even argue that relational spaces are still poignantly underrepresented in Jewish studies.^38^ For example, Julia Brauch, Anna Lipphardt and Alexandra Nocke define Jewish places as everything that is geographically located and thus belongs to a certain location that is somehow associated as Jewish, while Jewish spaces are linked to performance.^39^ From this point of view, urban historical studies have investigated Jewish/non-Jewish negotiations at physical sites associated with modern Jewish life, such as synagogues, institutional buildings, and cemeteries.^40^ Moving towards a more relational approach, Charlotte Elisheva Fonrobert and Vered Shemtov conceptualise Jewish spaces and the practices they elicit as production surfaces of politics and society, thus emphasising their communicative function.^41^ Together with Barbara E. Mann, they underline the untenability of a binary between Jewish places and Jewish spaces, implicitly suggesting that a Tuanian approach that links social relations and material structures better suits the historical Jewish experience. Still, the role of physical attributes in shaping Jewish/non-Jewish relations largely remains elusive.

Joachim Schlör sketches a possible approach with help from migration history. Jewish movement between countries or houses, he argues, always requires a ‘“doorstep,” a *limen*, on which farewells and greetings, friendly or unfriendly, are exchanged’.^42^ While Schlör employs a flexible and metaphorical definition of the doorstep, he also highlights how its material dimension becomes ‘the very point where […] constructions, assumptions, negotiations, and representations of identity and difference take place’ as Jews *do* their lives.^43^ The doorstep, as any other border, should not be viewed as a line of division but rather as a location of transgression, thus shaped by (non-)contact and exchange.^44^ In emphasising doorsteps to Jewish homes, as well as synagogue inscriptions and *eruvim* that too are positioned in-between the public and the private, as spaces for Jewish/non-Jewish encounters, Schlör guides the field’s overwhelming focus on interaction, contact zones and spheres of exchange, shared presence, and/or a cultural negotiation of Jewishness towards material forms.^45^ Building upon his thesis, we want to remedy the lack a theoretical mooring in how material structures define the nature and aspects of Jewish/non-Jewish mingling.^46^ Liminality, as Schlör suggests, offers a fruitful path forward.

In the 1960s, Victor Turner anchored Arnold van Gennep’s original processional and transitory understanding of liminality in concrete rituals.^47^ Employed in a variety of disciplines to study and explain the ambiguous, disoriented human experience of being in movement, in transit, or in a moment of transformation, Les Roberts comments that liminality is more often than not linked to Lefebvre’s ‘largely rhetorical space of flow, hybridity and becoming’,^48^ rather than absolute space. Nevertheless, scholars in urban studies are at the forefront of anchoring liminality in material structures such as streets, porches, hallways, doors, balconies, and windows. These places are concretely situated in-between public and private realms, thus facilitating transitory movements and temporary interactions. Defining ‘urban liminal architecture’ as ‘operative’, Carolin Aronis argues that material in-between openings facilitate an ‘ephemeral, transient moment’ of communication with the other.^49^ Following her relational approach, we understand urban liminal topographies to be unregulated by the norms of the public/private dichotomy, thus creating a space of ‘connectivity through the imagined communication, through a potentiality of interaction’.^50^ Thus, the materiality of liminal places not only shape but in themselves create relational spaces, in which urban inhabitants run the potential to momentarily encounter each other in an undefined atmosphere of vulnerability and safety, as well as separation and freedom.^51^ It is precisely this interdependence between inside and outside, private and public, that draws Schlör towards thresholds. In conceptualising doorsteps as transitional spaces, he understands them as creators of the tension between ‘longing and belonging’ as it is produced in social negotiations, in both modern urban Jewish life as well as historically more distant periods.^52^ What is more, the urban scholarly understanding of liminal topographies is anchored in the collapsed space/place duality: The very material structure facilitates both cultural meanings and transitory social interactions, which in turn develop the continued shape and use of the physical place.

Despite its potential in aligning absolute space with relational space, liminality as a concept and a theoretical approach remains largely abstract and unconceptualized in Jewish studies.^53^ Research interested in liminal spaces is largely confined to the state-of-being as suspended in a collapsed duality, as an experience of the uncertain and undefined terrain between two opposing, metaphysical poles, thus missing liminality’s relational dimension.^54^ We wonder, perhaps provocatively, if the use of the concept of space, and that of liminal space in particular, has become mainly an application of a trendy word, rather than an in-depth engagement with its theoretical ramifications. With this in mind, we turn to our two case studies: A staircase leading up to an orthodox synagogue in Stockholm, and a corridor in an apartment house in Vienna. We approach these material structures as liminal topographies. They were material structures situated in-between private and public spheres with a high potential for facilitating everyday encounters between Jews and non-Jews. The staircase and the corridor became undefined, transitory shared spaces, and thus, the social interactions they expediated existed outside of societal norms, holding potential for a variety of Jewish/non-Jewish relations.

## Everyday meetings: the staircase of *Adass Jisroel* in Stockholm

At the turn of the twentieth century, Stockholm’s orthodox synagogue *Adass Jisroel* was housed in a Pietist girl orphanage. The synagogue had most likely been founded in response to the construction of the Great Synagogue in the city centre in 1870, which marked the Jewish community’s official side-lining of orthodox practices.^55^ In 1871, the rental of a school venue on the first floor in the Pietist orphanage on *Sankt Paulsgatan* 17 was secured for Jewish orthodox practices, marking the start of a Jewish/Christian co-shared space. The Pietist orphanage, which had renovated the building according to their practical needs and religious motivations in 1859 prior to the synagogue’s rental of the school venue,^56^ ultimately shaped the orthodox minority’s experience of being Jewish in Protestant Stockholm. A staircase played a vital role in defining Jewish/Christian everyday interactions.

**Figure 1.**
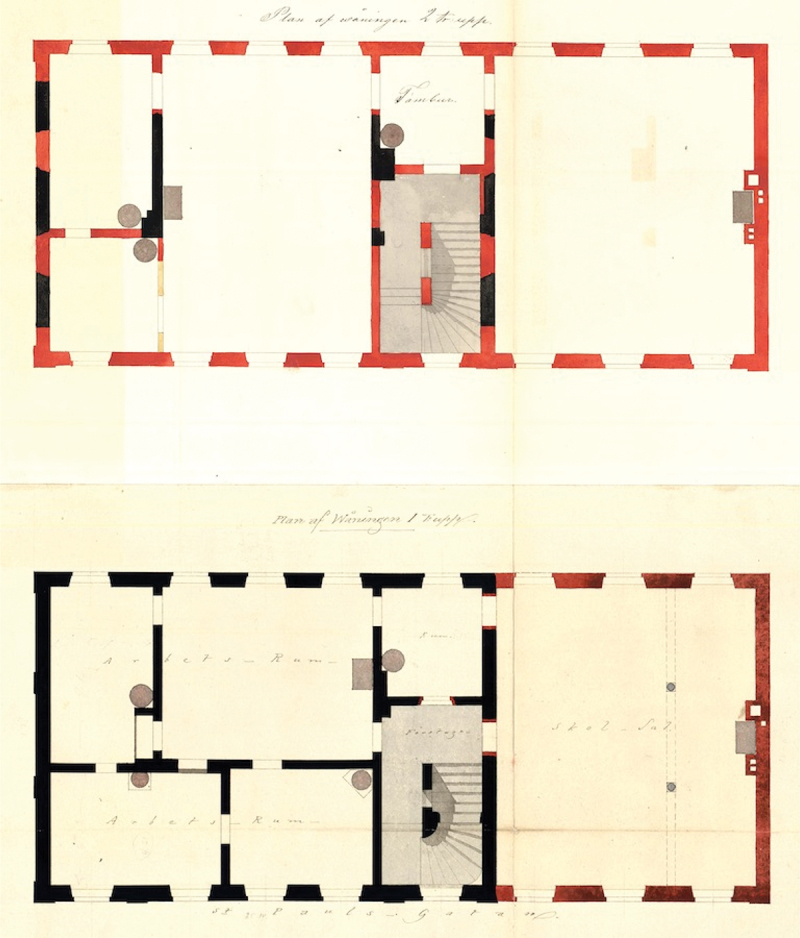
Plans of second and first floors of the pietist orphanage from 1859. Stockholm’s older building designs, *Stockholms stadsarkiv*.

The staircase, our first example of a liminal topography, was located in-between public and private domains. It could only be accessed by opening the front door to the building and stepping over the threshold, exiting the public street. At the same time, while hidden behind walls and doors, Figure 1 shows that its central architectural position elicited a transportation of orphans, orphanage personnel, and orthodox Jews alike throughout the building, leading the latter to the privacy of their synagogue on first floor. The weekly rituals performed in the synagogue included morning and sometimes evening prayers,^57^ meaning that the group visited the building at least once a day. The staircase’s lack of stoves did not support longer interactions, especially during cold winters, but it connected people in movement before diverting them into different activities in different rooms. We do not know the exact nature of this contact: Primary sources are silent about the subjective experience of shuffling up and down the staircase. We do not know if its use was meticulously scheduled, with Jews, female orphans, and orphanage personnel moving up and down at different hours during the day, or if movements occurred more freely, prompting communicative gestures and perhaps even conversations. What we do know is that the staircase’s function as an architectural aorta meant that Jews and non-Jews came in close bodily contact on a daily basis. Facilitating movements that encouraged transitory interactions in the residential home of Pietist orphans, the staircase functioned as a space of in-betweenness that, contrary to the depiction of orthodox life in Swedish historiography, suggests a connection – a relationship – between two disparate urban groups.

In zooming in on the architectural design of the building that housed *Adass Jisroel*, we answer Martyna Bryla’s call that scholars need to move beyond in-betweenness as a theoretical condition and focus on ‘what *happens* in the interstices and what it may tell us about the people, places or cultures involved’.^58^ Similarly, Jewish urban studies have, through what we conceptualize as a topographical understanding of liminal spaces, taken a small but significant step towards an engagement with the relational attribute of liminality, exploring Jewish/non-Jewish interactions through and in material openings in-between public and private realms. For example, Dana E. Katz argues that windows in the sixteenth century’s Venetian ghetto operated as physical sites for the construction of social boundaries between citizens of the Christian city state and its religious outsiders: Jews. As ‘mediators’ and ‘barriers’, these material perforations in the walls of ghetto houses were blocked or barred, establishing the Jewish community as a spatial and societal void, removed from the ‘optical powers’ to gaze at, and thus participate in, the Christian world.^59^ Liminal spaces as sites for the civic navigation of religious and ethnic power relations is also touched upon in Maté Rigó’s study on superintendents of yellow-star houses in Second World War-Budapest. With their apartments located at house entrances, and with sole responsibility of the key to the house, superintendents and Jewish inhabitants developed relationships that ventured beyond that of ‘victimizer-victimized’.^60^ Clearly, the material design of urban environments impacts the intensity of Jewish/non-Jewish relations.

While the intensity of the relationship between Pietist orphans and orphanage personnel and orthodox Jews in Stockholm eludes us, we can get closer to the relational aspect of liminal topographies by digging deeper into the sensory experience that the staircase would have created. Even if intersections between Jewish and Pietist bodies were planned to a minimum, the material quality of the staircase would have perforated such a division anyway. Upon visiting the synagogue in 1905, a reformed Jewish journalist mentioned that prayers and conversations could be heard through the door and that Jewish children ran ‘out of and into the venue’ throughout the *shabbat* service.^61^ While the journalist’s visit took place after the Pietist orphanage had moved out of the building, his testimony testifies to the porous material border between the room used by the Jewish group and the employees and children working and living in the rest of the building. As a liminal topography, the staircase ensured continuous sensory interactions between orthodox Jews and non-Jews via sound and (potentially) touch, and emphasised the orthodox group’s bodily immersion into the non-Jewish world. Located within the Pietist orphanage, literally just a wooden door away from its permanent residents, *Adass Jisroel*’s venue underlined the synagogue’s belonging to Stockholm’s urban society and enforced on-going interactions with the Pietist community. At the turn of the twentieth century, and contrary to historiographical claims, Stockholm’s orthodox Jews were not reclusive. Instead, their daily life was practiced in an environment that was defined by its bodily and sensory vicinity to non-Jews, which increased potential for Jewish/non-Jewish interactions.

## Shared Jewish/non-jewish spaces: corridors in residential spaces in Vienna

As demonstrated through the case study of Stockholm, we seek to answer Löw’s call for a more focused approach on the relational attributes of space by introducing liminal topographies. In this second example, we deepen the relational dimension of liminality by approaching it as a ‘shared space’ for Jews and non-Jews.^62^ Liminality urges us to not think of private and public spaces as distinct and separate entities, and this resonates with the concept of cohabitation, popularized by Paul Gilroy. Gilroy found that people of ethno-cultural heterogenous backgrounds mostly live together peacefully, and he developed the notion of ‘daily interactions’ that happen repeatedly due to scenarios of co-existence, reminding us to investigate how people living together shape the many small daily interactions they share.^63^ Similarly, the concept of conviviality provides a useful approach of how to think of modes of human togetherness yet keep focus on ‘living with differences’.^64^ With these four concepts at hand, we seek to move beyond the Jewish/non-Jewish everyday encounters that the staircase in Stockholm revealed and look even deeper into the human relationships that developed in a liminal topography due to daily, ongoing meetings.

During such meetings, recognition of ‘similarity’ towards peers was more commonly experienced as part of navigating daily interactions.^65^ In addition to conventional analytical perspectives, such as Jewish ‘difference’,^66^ this concept helps considering Jewish/non-Jewish relations in all its shapes and forms, ranging from good to bad. Furthermore, it provides access points to everyday, non-choreographed encounters between Jews and non-Jews.^67^ In line with this development, we propose that the concept of liminality allows researchers to focus on the role of material structures in facilitating spaces that prompted not only interactions, but relationships. While Benjamin’s quote in the introduction referred to the loggia as a somewhat aloof and estranged architectural structure in-between public and private life, and thus belonging to neither and both, we see liminal topographies as operative in their own right. They exist as a spatially unspecific and uncategorized merging of private and public spheres, and are thereby endowed with potential and choice for urban agents. In short, they are socio-spatial, absolute topoi for the development of transitory but ongoing, everyday Jewish/non-Jewish relations.

This brings us to an extension of the previous qualitative, digital analysis of the Viennese Jewry. In the autobiography *Glockengasse 29*, author Vilma Neuwirth (née Kühnberg, 1928–2016) particularly underlines liminal topographies as arenas of permanent contact. She presents the apartment building in general, and the corridor at *Glockengasse* 29 in *Leopoldstadt* in particular, as an important meeting and exchange space between Jews and non-Jews. In her memoir, Neuwirth recounts her childhood in the 1920s and 1930s. She repeatedly accentuates that there was intensive contact among the residents of the apartment house. She emphasises that this contact was defined by ‘good neighbourly relations’ and many friendships. According to Neuwirth’s memories, the architectural realities of overcrowding forced most families to live not so much in their flats but rather in the whole building:

Mr and Mrs Högenwarth, who were my father’s customers, also lived on our floor. We had a particularly good neighbourly relationship with them. […] The doors of the apartments were not locked; you came and went to your neighbors as you pleased.^68^

What resonates even stronger is Neuwirth’s memory of the corridor in her apartment building, which, like Benjamin’s reminiscences of the loggia, connected private and public spheres. It was an important contact zone for residents of the house:

We had such a warm atmosphere in the corridor that everyone knew everything about each other. In summer, when it was extremely hot, it became especially cozy. The women appeared at the gangway with a bucket or bowl and an armchair. They filled the respective vessels with water and sat down, having their feet in the cool water, in front of the windows belonging to their apartments. Everyone had their coffee mug with *Zichorienkaffee* [chicory coffee; a coffee substitute]. They would sit there for hours, gossiping and joking.^69^

The cramped rooms, which did not include sanitary facilities, seemed uninviting, especially during summer. The apartments were warm and prone to a smell contaminated by excretory odours. The corridor, on the other hand, with its air circulation and shady corners, literally lured residents into the common areas of the apartment building. Here they sat together and shared pleasantries, enjoying a cold footbath filled with water that they fetched from the common tap. Neuwirth’s portrayal of the social dimension of the corridor resonates with new findings of daily encounters between Jews and non-Jews in urban spaces, particularly in relation to the distinct ambience that liminal topographies provide. When meeting in liminal *and* shared spaces, such as corridors, Hödl found that people tended to draw attention to former experiences, relationships, and meaningful contacts which they shared with the other in order to mingle and have a good time together.^70^ But what makes the special character of such spaces? How did they foster relationships instead of mere encounters or co-presence under crowded living conditions?

The apartment building in which Neuwirth grew up was located in the very heart of the district renowned as the ‘Jewish quarter’. The Kühnberg family lived in a neighbourhood full of shops, coffeehouses, theatres, and religious sites, just two blocks away from the southeast end of Augarten, a large public park, and a five-minute walk to the Prater, the place where people spent their leisure time. *Taborstra ße*, one of the city’s most famous vaudeville areas, was located just across their block. Also, the Leopoldstätter Tempel, the largest synagogue of Vienna, was only a ten-minute walk away. Many Catholic churches and smaller Jewish prayer rooms were found on or across the street. And, literally just a few doors down the block, they would have found the famous Varieté Reklame, a cinema and vaudeville that hosted the most popular performances at the time.^71^

**Figure 2.**
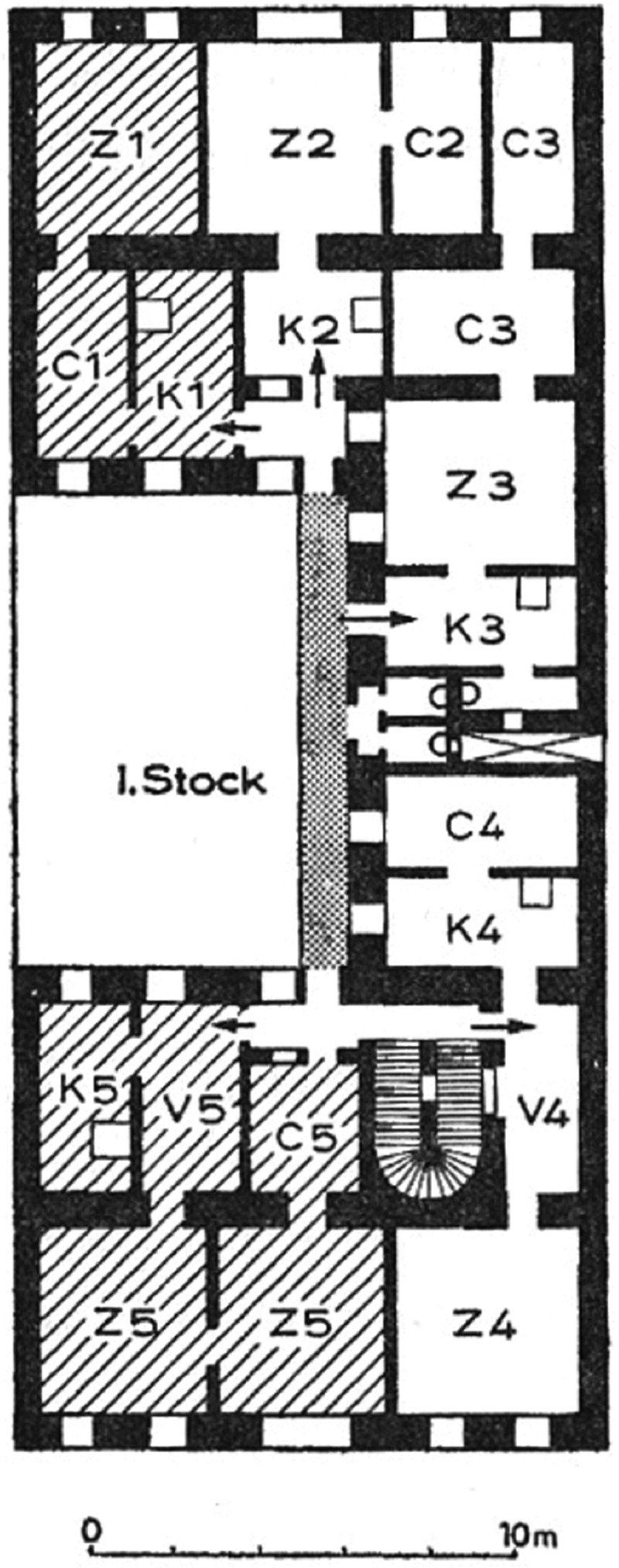
The typical *Zimmer, Küche, kabinett* (in the plan C1, K1 and Z1), Hans Bobeck and Elisabeth Lichtenberger, *Wien Bauliche Gestalt und entwicklung seit der Mitte des 19. Jahrhunderts* (Vienna: 1978), 69.

It was here, at *Glockengasse* 29, that Neuwirth grew up in a Jewish-Catholic family. Her father had immigrated to Vienna from Budapest, the second residential city of the Habsburg Empire, with three children from his first marriage. He met Vilma’s mother, a domestic worker from a rural area in Lower Austria, in Vienna. Only a few years later, the working-class couple had eight children to care for. Vilma’s father, Joseph Kühnberg (1888–1942), was a hairdresser with his own business, and her mother, Maria Kühnberg (née Böhm, 1892–?), took care of the children and her parents-in-law. How did a family of 10 live in Vienna? Or to put it more aptly: Who shared the living space commonly imagined as a private apartment? The Kühnberg family settled in a typical *Zimmer, Küche, Kabinett* (room, kitchen, and connecting room) apartment, see Figure 2. This means that the family shared an apartment of approximately 50 square meters, which included access to two separate toilet areas, with no running water. Furthermore, due to the massive population growth in Vienna, the vast majority of its inhabitants had to share housing with their fellow citizens. This led to as much as 20 per cent of the population ending up as *Bettgeher* (bed lodgers, or people who could not even afford to rent a shared room and had to pay for only a bed to sleep in).^72^ This means that up until the 1930s, one fifth of Vienna’s inhabitants shared the allegedly most intimate atmosphere of a home with an average of six up to 10 other non-family members. Accordingly, there existed plenty opportunities for Jewish/non-Jewish relations to emerge in private spaces.

The building at *Glockengasse* 29 had three floors. Kühnberg’s hairdressing salon and a grocery store was located on ground floor. Ten residential units were divided between first and second floors, while two residential units were located under the roof, on third floor. The grocery store next to Kühnberg’s salon was run by the Bergkirchner family. On first floor, the Vanetscheks and the Häuslers, a non-Jewish and a Jewish family respectively, had their apartments. The Kühnbergs lived on second floor with Mr and Mrs Högenwarth, the Novotny family, and a coffeehouse owner named Ms Kemper. Under the roof, Hirsch David and his wife and a non-Jewish family had their apartments. The apartments were oriented towards the street and the courtyard. The corridor was arbour-like and led towards the entrances of the apartments. As is apparent already in the quoted passages above, these liminal topographies functioned as shared spaces. While religious and cultural identifications became meaningless in the housing environment, those gathering in the corridors belonged to the same class. The housing situation was a shared experience; one in which class trumped other affiliations.

The importance of such shared spaces is highlighted by the research group of the New York Tenement Museum. Questioning the dynamic between city residents – migrants and non-migrants, Jews and non-Jews, and among other groups alike – they emphasized the distinct importance of balconies and staircases in providing the most productive ground for interactions among neighbours and the formation of relationships. As they learned from searching through the bulk of testimonies in their archive, balconies and staircases were open areas where inhabitants of a building met, functioning as zones of active engagement with one’s neighbours.^73^ These shared spaces are also attested to be of particular importance in studies on inter-ethnic conviviality and the question of how diverse forms of neighbourliness are created and maintained. Tilman Heil, for example, attests that these transitional areas ensure the balance between situations of co-operation and conflict when living together.^74^ Thus, in the balcony corridor at *Glockengasse* 29, people from different social and political backgrounds were made to mingle. Neuwirth’s depictions quoted earlier illustrate the scale of intensity that meetings in liminal spaces could assume: The relationship between people sharing the same home address could range from mere encounters in the courtyard to lively interactions in the corridor in the form of shared meals or coffee chats.

## Liminal topographies in the study of Jewish/non-jewish relations: a relational approach

In this article, we have assessed liminal topographies in relation to everyday spaces in European metropolises at the turn of the twentieth century and defined them as relational products. We argue that only by doing so, is it possible to get closer to the intensity scale of Jewish/non-Jewish relations, especially among the broader masses. We have demonstrated that this spatial focus expands conventional historiographical narratives, revealing them as insufficient or even revising them completely. The exploration of liminal topographies in Stockholm shows that orthodox Jewish life was embedded in an urban environment that carried a high potential for everyday relations with non-Jews: Orthodox Jews did not cluster in specific neighbourhoods, religious practices moved them across the whole city, and the staircase leading up to their synagogue ensured bodily interactions with orphans and orphanage personnel. The presumed historiographical narrative of a spatially and socially isolated group of Eastern European and orthodox Jews therefore does not hold. Similarly, although perceived as a ‘Jewish’ neighbourhood, the district of *Leopoldstadt* in Vienna was home to both Jews and non-Jews. The digital mapping and textual analysis of autobiographies underline the liminal topography of apartment corridors as not only spaces of Jewish/non-Jewish mingling, but as shared spaces that fostered ongoing relationships across ethnic and religious borders. While the relationship between variables such as class, gender, age, sexuality, and race, as well as the nature of liminal topographies, needs further study, the current results prompt us to re-assert the field’s understanding of Jewish/non-Jewish sociability in modern European cities.

We understand that this is a provocative statement. The field of Jewish spaces and places has, after all, been established as a fruitful way to explore Jewish relationships to non-Jewish societies. As we have demonstrated, the field, however, lacks an in-depth engagement with the theoretical ramifications of the material dimension, particularly regarding Jewish/non-Jewish relations. We have sought to launch one possible avenue into considering space as more than a sheer label. The concept of liminal topographies helps directing the scholarly gaze towards architectural structures that both enabled and shaped different intensities of Jewish/non-Jewish relations on an everyday, daily basis, both among Jewish groups and in Jewish neighbourhoods commonly asserted as insulated. The small pool of previous studies even suggests that liminal topographies have played an important role for Jewish/non-Jewish relations in such disparate historic times as the Middle Ages and the very moments before deportations to death camps during the Second World War. Here, further research is needed. Should we see liminal topographies as a modern urban phenomenon, and if not, how does temporality affect the existence, use, and meaning of liminal topographies? What social, cultural, political, and environmental factors influence their creation – and dismantlement? And while we have shown that liminal topographies provide a methodological entry point to transnational comparisons of Jewish/non-Jewish relations, further case studies would shine light on geographical and societal nuances in its operational function. We think that research in this direction would have a strong impact on Jewish Studies since this article has shown that an engagement with attributes of absolute space can dismantle master narratives of Jewish isolation and push historical research towards a relational approach.

And yet, for Stockholm and Vienna, Jewish/non-Jewish relations facilitated by liminal topographies encountered limits as the twentieth century moved towards the 1930s. In Stockholm, *Adass Jisroel* relocated in 1917 and a thorough renovation made sure to seclude the entrance from non-Jewish bodies.^75^ Through further elimination of liminal topography, orthodox Jewish practice was slowly but surely removed from Stockholm’s public sphere. In Vienna, moving a few years forward in Vilma Neuwirth’s autobiography, less idyllic memories are revealed. As in many other autobiographies that report on life in the interwar period, the drastic changes that the seizure of power by National Socialism brought upon the perceived continuous and positive Jewish/non-Jewish relations become apparent. Concerning, for instance, the very next-door neighbours of the Kühnbergs, Neuwirth wrote: ‘Mr and Mrs Högenwarth, with whom we had lived together in best harmony for years, became mortal enemies overnight in 1938.’^76^ Or: ‘The Vanitscheks also made our lives hell during the Hitler years.’ And concerning her friends: ‘Our friends, whom we had known since we were little children and with whom we were together every day, insulted us in the meanest way.’^77^ Such findings on the transformation of Jewish/non-Jewish relations under National Socialism are, of course, not new. Why, then, is the spatial homogeneity of Jewish neighbourhoods important to question at all, and what needs to be further done to better understand Jewish spatial practices and relations in Europe before the Holocaust?

First, our work demonstrates that conventional interpretations of antisemitism are somewhat outdated and need to be expanded. Studies on the process of Jews acquiring bourgeois equality have highlighted that it was the exclusion of the Jewish population from associations from the turn of the twentieth century onward, and the supposedly fewer contacts between Jews and non-Jews, that nourished an increasing radicalization of antisemitism in the first decades of the twentieth century.^78^ However, after our investigation of the housing conditions and religious institutions of the Jewish working class, a narrative of isolation cannot be sustained for neither Jewish nor non-Jewish neighbourhoods. The revised narrative, which goes beyond dichotomous conceptions of Jewish/non-Jewish relations, helps questioning the presumption of Jewish exclusivity. With this new paradigm in mind, it is important to learn more of how it could be that such frequent and positive exchanges did not lead to lasting relationships in the face of National Socialism, and how exclusion mechanisms were implemented so radically within such a short time.

To answer the second question, we believe that the tools and techniques that continue to emerge as part of Digital Humanities promises new ways to explore and combine qualitative and quantitative analysis towards relational insights. While our investigation in this article provides an embryonic design of what such a study might look like within the spatial approach – moving as it does between cartographic macro geographies and liminal microstructures – recent developments offer further approaches that hone methodological creativity and flexibility.^79^ In line with this, we wish future research to introduce approaches, tools, and techniques that can map liminal topographies as deep relational products. We are convinced that a combined material and digital approach would further help the field to better understand the variety of Jewish/non-Jewish relations among the broad masses of urban inhabitants.

## Notes

1. The premise of this article developed from our joint participation in the panel ‘Reconsidering the spatial turn in Jewish studies: A relational approach’ at the POLIN conference *What’s New, What’s Next? Innovative Methods, New Sources, and Paradigm Shifts in Jewish Studies* on 3–7 October 2021. The article also owes to our two babies who were born at the end of 2022. Their support – entering this world timely, and then being great sleepers – was invaluable to their mothers. We are also indebted to Joachim Schlör: Not only has his vast body of work been a continuous inspiration for us, his never-ending support of our own work has also been crucial to get this article published. Finally, our argument has been greatly improved by generous suggestions from two anonymous peer-reviewers. All translations are ours.

2. Walter Benjamin, *Berlin Childhood around 1900* (Cambridge/London: The Belknap Press of Harvard University Press, 2006 [1950]), 42.

3. Heide Imai, “The liminal nature of alleyways: Understanding the alleyway roji as a “boundary” between past and present,” *Cities* 34 (2013): 58–66; Hugh Matthews, “The street as a liminal space: the barbed spaces of childhood,” in *Children in the City: Home, Neighbourhood and Community*, eds. Pia Christensen, and Margaret O’Brian (London/New York: Routledge, 2003), 101–17.

4. In 1910, 54 per cent of the Viennese population was not born in the city. Michael John, ‘Vielfalt und Heterogenität: Zur Migration nach Wien um 1900,’ in *Migration und Innovation um 1900: Perspektiven auf das Wien der Jahrhundertwende*, ed. Elisabeth Röhrlich (Wien/Köln/Weimar: Böhlau, 2016), 23–64, here 27.

5. Ibid., 39.

6. Ivar Oxaal, “Die Juden im Wien des jungen Hitler: Historische und soziologische Aspekte,” in *Eine zerstörte Kultur: Jüdisches Leben und Antisemitismus in Wien seit dem 19. Jahrhundert*, eds. Gerhard Botz, Ivar Oxaal, Michael Pollak, and Nina Scholz (Wien: Czernin, 2002), 46–64, here 52.

7. Carl Henrik Carlsson, *Medborgarskap och diskriminering: Östjudar och andra invandrare i Sverige, 1860–1920* (Uppsala: Acta Universitatis Upsaliensis, 2004), 31.

8. Anna Besserman, ““ … Eftersom nu en gång en nådig försyn täckts hosta dem upp på Sveriges gästvänliga stränder”: Mosaiska Församlingen i Stockholm inför den östjudiska invandringen till staden, 1860–1914,” *Nordisk Judaistik* 5, no. 2 (1984): 13–38, here 29.

9. Svante Hansson, *Flykt och överlevnad: Flyktingverksamhet i Mosaiska församlingen i Stockholm, 1933–1950* (Stockholm: Hillelförlaget, 2004), 44; Joseph Zitomersky, “The Jewish Population in Sweden, 1780–1980: An Ethno-Demographic Study,” in *Judiskt liv i Norden*, eds. Gunnar Broberg, Harald Runblom, and Mattias Tydén (Uppsala: Acta Universitatis Upsaliensis, 1988), 99–125, here 114; “Norrjudar och söderjudar: Södermalm,” Stadsmuseet, accessed March 3, 2021, https://www.stadsmuseet.stockholm.se/kalendarium/2021/03/20/norrjudar-och-soderjudar-sodermalm/.

10. One exception is: Mats Franzén, *Den folkliga staden: Söderkvarter i Stockholm mellan krigen* (Lund: Arkiv förlag, 1992), 144–54.

11. Susanne Korbel, “Spaces of Gendered Jewish and Non-Jewish Encounters: Bed Lodgers, Domestic Workers, and Sex Workers in Vienna, 1900–1930,” *Leo Baeck History Yearbook* 65 (2020): 88–104, here 90.

12. Marsha Rozenblit, *The Jews of Vienna, 1867–1914: Assimilation and Identity* (New York: State University of New York Press, 1983), 71.

13. Oxaal, “Die Juden im Wien,” 52–3; Klaus Hödl, *Entangled Entertainers: Jews and Popular Culture in Fin-de-Siècle Vienna* (New York, Oxford: Berghahn 2019).

14. See, for example, these otherwise excellent studies: Karen Auerbach, *The House at Ujazdowskie 16: Jewish Families in Warsaw after the Holocaust* (Bloomington/Indianapolis: Indiana University Press, 2013), 72; Elisabeth Anthony, *The Compromise of Return: Viennese Jews after the Holocaust* (Detroit: Wayne State University Press, 2021), 12.

15. Maja Hultman, “The GIS prism: Beyond the myth of Stockholm’s Ostjuden,” in *Jewish Studies in the Digital Age*, eds. Gerben Zaagsma, Daniel Stökl Ben Ezra, Miriam Rürup, Michelle Margolis, and Amalia S. Levi (Berlin/Boston: De Gruyter Oldenburg, 2022), 125–45; Maja Hultman, “Between marginality and multiplicity: Mapping Jewish public home-making in modern Stockholm,” (University of Southampton, PhD Thesis, 2019), 46–50.

16. Gerben Zaagsma, “#DHJewish – Jewish Studies in the Digital Age,” *Medaon* 23 (2018): 1–11.

17. Alberto Giordano, and Tim Cole, “The limits of GIS: Towards a GIS of place,” *Transactions in GIS* 22, no. 3 (2018): 664–76; Mary Anne Poutanen, and Jason Gilliland, “Mapping Work in Early Twentieth-Century Montreal: A Rabbi, a Neighbourhood, and a Community,” *Urban History Review* 45 (2017): 7–24; Gethin Rees, Nicholas de Lange, and Alexander Panayotov, “Mapping the Jewish Communities of the Byzantine Empire using GIS,” in *Migration and Migrant Identities in the Near East from Antiquity to the Middle Ages*, eds. Justin Yoo, Andrea Serbini, and Caroline Barron (New York: Routledge, 2019), 100–18; Marcin Wodzinski, *Historical Atlas of Hasidism* (Princeton: Princeton University Press, 2018).

18. The mapping is based on data compiled from the Jewish community’s membership lists at *Riksarkivet*, and Stockholm City’s taxation lists and lists of ‘alien faith confessioners’ at local church parish records at *Stockholms stadsarkiv*. The spatial information was mapped with the software programme ArcGIS, using Alfred Bentzer’s city map from 1909, also available at *Stockholms stadsarkiv*.

19. For an in-depth discussion, see: Hultman, “The GIS prism,” 132–136.

20. The map is based on open street map and GIS information provided by the city of Vienna. We visualized the map using the spatial analysis packages folium, geopandas and leaflet in Python.

21. We are inspired by Alberto Giordano’s and Tim Cole’s network analysis study of the 1944 Nazi ghetto and collective housing in Budapest. Giordano and Cole included space but also a temporal dimension, namely the average walking speed and time for covering distances, to analyze what contact possibilities existed under extreme repressive measures: Alberto Giordano, and Tim Cole, “On Place and Space: Calculating Social and Spatial Networks in the Budapest Ghetto,” *Transactions in GIS* 15, no. 1 (2011): 143–70.

22. Ruth Beckermann, *Die Mazzesinsel: Juden in der Leopoldstadt 1918–1938* (Vienna: Löcker, 1992).

23. It has to be noted that not even people in better-off households lived exclusively with family members. It was common for the middle and upper classes to employ domestic servants who lived with the families they worked for: Korbel, “Spaces of Gendered Jewish and Non-Jewish Encounters,” 3–10.

24. Travor M. Harris, “Deep Geography – Deep Mapping: Spatial Storytelling and a Sense of Place,” *Deep Maps and Spatial Narratives*, eds, David J. Bodenhamer, John Corrigan, and Trevor M. Harris, eds., (Indianapolis: Indiana University Press, 2015), 28–53.

25. Martina Löw, and Gunter Weidenhaus, “Borders that relate: Conceptualizing boundaries in relational space,” *Current Sociology* 65, no. 4 (2017): 553–70.

26. Doris Bachmann-Medick, *Cultural Turns: Neuorientierungen in den Kulturwissenschaften* (Hamburg: Rowohlt, 2014), 285.

27. Ibid., 285–318.

28. Michel de Certeau, *The Practice of Everyday Life* (Berkeley: University of California Press, 1984), 110–18.

29. Henri Lefebvre, *The Production of Space*, (Oxford, Cambridge: Blackwell, 1991), 26–7. On the social construction of spaces see also: Martina Löw, *Raumsoziologie* (Frankfurt am Main: Suhrkamp, 2015), 63–8.

30. Martina Löw, “O spatial turn: para uma sociologia do espaço,” *Empo social: revista de sociologia da USP* 25, no. 2 (2013): 17–34.

31. Martina Löw defines this dichotomous categorization as absolutist and relativist understanding of space. Martina Löw, “The Constitution of Space: The Structuration of Spaces Trough the Simultaneity of Effect and Perception,” *European Journal of Social Theory* 11, no. 1 (2008): 25–49, here 26.

32. Lefebvre, *The Production of Space*, 57.

33. Michel de Certeau, “Praktiken im Raum,“ in *Raumtheorie: Grundlagentexte aus Philosophie und Kulturwissenschaften*, eds. Jörf Dünne, and Stephan Günzel (Frankfurt am Main: Suhrkamp, 2015), 343–53, here 345–47.

34. Barbara E. Mann, “Space and Place,” in *The Routledge Handbook of Contemporary Jewish Cultures*, eds. Laurence Roth, and Nadia Valman (London/New York: Rutgers University Press, 2015), 183–94; Arijit Sen and Lisa Silverman, eds., *Making Place: Space and Embodiment in the City* (Bloomington: Indiana University Press, 2014).

35. Yi-Fu Tuan, *Space and Place: The Perspective of Experience* (Minneapolis: University of Minnesota Press, 2001), 6.

36. Julia Brauch, and Anna Lipphardt, “Gelebte Räume – Neue Perspektiven auf jüdische Topographien,” in *Jewish Spaces: Die Kategorie Raum im Kontext kultureller Identitäten*, eds. Petra Ernst, and Gerald Lamprecht (Innsbruck/Wien/Bozen: Studienverlag, 2010), 13–32, here 19–22; Susanne Korbel, Lukas Nievoll, and Thomas Stoppacher, eds., Special issue: Rethinking Jewish and non-Jewish Relations, *Jewish Culture and History* 21, no. 1 (2020); Simone Lässig, and Miriam Rürup, eds., *Space and Spatiality in Modern German-Jewish History* (New York: Berghahn, 2017); Barbara E. Mann, *Space and Place in Jewish Studies* (Piscataway: Rutgers University Press, 2012), 2–3.

37. Mann, *Space and Place in Jewish Studies*, 17–8; at the same time, the special issue by Michael Miller and Erika Szivós is an excellent example of the entrance of the spatial turn in Jewish Studies: Michael Miller, and Erika Szivós, “Introduction: Historic Jewish Spaces in Central and Eastern European Cities,” *East Central Europe* 42 (2015): 139–62.

38. Simone Lässig, and Miriam Rürup, “Introduction: What Made a Space “Jewish”?: Reconsidering a Category of Modern German History,” in *Space and Spatiality in Modern German-Jewish History*, eds. Lässig and Rürup, 1–20, here 8.

39. Julia Brauch, Anna Lipphardt, and Alexandra Nocke, “Exploring Jewish Spaces,” in *Jewish Topographies: Visions of Space, Traditions of Place*, eds. Julia Brauch, Anna Lipphardt, and Alexandra Nocke (Burlington, Hampshire: Ashgate, 2008), 1–23, here 4.

40. See, for example: Saskia Coenen Snyder, *Building a Public Judaism: Synagogues and Jewish Identity in Nineteenth-Century Europe* (Cambridge: Harvard University Press, 2013); Marion Kaplan, *Hitler’s Jewish Refugees: Hope and Anxiety in Portugal* (Yale: Yale University Press, 2020); Davin E. Naar, *Jewish Salonica: Between the Ottoman Empire and Modern Greece* (Stanford: Stanford University Press, 2016).

41. Charlotte Elisheva Fonrobert, and Vered Shemtov, “Introduction: Jewish Conceptions and Practices of Space,” *Jewish Social Studies* 11, no. 3 (2005): 1–8, here 2.

42. Joachim Schlör, “Faith in Residence. Jewish Spatial Practice in the Urban Context,” in *Space and Spatiality in Modern German-Jewish History*, eds. Lässig, and Rürup, 232–45, here 232.

43. Schlör, “Faith in Residence,” 236.

44. Joachim Schlör, ““Ach, man läßt mich durch. Es ist gelungen”. Die Überschreitung der deutschen Grenze in Emigrationsberichten,” in *Jüdischer Almanach der Leo Baeck Institute: Grenzen*, ed. Gisela Dachs (Frankfurt: Jüdischer Verlag im Suhrkamp Verlag, 2015), 95–105, here 100.

45. Susanne Korbel, *Auf die Tour! Jüdinnen und Juden in Singspielhalle, Varieté und Kabarett. Zwischen Habsburgermonarchie und Amerika* (Vienna: Böhlau, 2021), 54; Hanna Kozinska-Witt provides an example of an investigation of Jewish and Christian neighborhoods: Hanna Kozinska-Witt, “Juden in den Städten oder zwei Fragen an die Stadtgeschichte,” in *Dekonstruieren und doch erzählen. Polnische und andere Geschichten*, ed. Jürgen Heyde (Göttingen: Wallstein, 2015), 261–67.

46. Alexandra Binnenkade offers one of few studies that emphasize that the materiality of spaces allows us to draw conclusions about their relational character: Alexandra Binnenkade, “Of Sounds and Stones. The Jewish-Christian Contact Zone of a Swiss Village in the Nineteenth Century,” in *Space and Spatiality in Modern German-Jewish History*, eds. Lässig, and Rürup, 23–39.

47. Victor Turner, “Betwixt and Between: The Liminal Period in *Rites de Passage*,” in *Reader in Comparative Religion: An Anthropological Approach*, eds. William Aarmand Lessa, Evon Zartman Vogt, and John Mamoru Watanabe (New York: Harper & Row, 1979 [1964]), 234–43.

48. Les Roberts, *Spatial Anthropology: Excursions in Liminal Space* (London: Rowman & Littlefield, 2018), 41.

49. Carolin Aronis, “Architectural liminality: the communicative ethics of balconies and other urban passages,” *Cultural Studies* 36, no. 3 (2022): 475–501, here 493.

50. Ibid., 495.

51. Ibid., 491, 493. “The analytical potential of liminal spaces is also emphasised in: Roberts,” *Spatial Anthropology*, 37.

52. Schlör, “Faith in Residence,” 233.

53. For example, when Center for Jewish Studies at the University of California Santa Cruz organised the conference *Liminal Spaces and the Jewish Imagination* in 2015, it employed a broad understanding of liminality, ranging from literary spaces to virtual landscapes: “Liminal Spaces and the Jewish Imagination,” University of California Santa Cruz, accessed August 5, 2022, https://www.cjs.ucsc.edu/2015/01/20/liminal-spaces-conference/.

54. Erica Smeltzer, “The Metropolis and the Attic: Spatial Representations of Jewish Identity in Kafka and the Golem of Prague,” *Partial Answers: Journal of Literature and the History of Ideas* 14, no. 2 (2016): 343–60. A notable exception is Schlör’s work, “Faith in Residence.” See also: Piera Rossetto, “Space of Transit, Place of Memory: Ma’abarah and Literary Landscapes of Arab Jews,” *Quest* 4 (2012): 103–27.

55. Maja Hultman, “The Construction of the Great Synagogue in Stockholm, 1860–1870: A Space for Jewish and Swedish-Christian Dialogues,” *Arts* 9, no. 1 (2020): 22.

56. For further analysis about the architecture of the Pietist orphanage, see: Maja Hultman, and Sophie Cooper, “Revisiting space and emotion: New ways to study buildings and feelings,” *History Compass* 21, no. 5: e12764.

57. Letter from Jacob Ettlinger to Arthur Fürstenberg, October 4, 1926, Personal Archive of Jacob Ettlinger, SE/RA/720483/5/8, Swedish State Archive.

58. Martyna Bryla, ““A Feast for the [Cold-War] Imagination”: Liminal Eastern Europe in the Writings of John Updike, Joyce Carol Oates and Philip Roth,” *European Review* 30, no. 4 (2022): 478–89, here 481. Original italics.

59. Dana E. Katz, ““Clamber not you up to the casements”: On ghetto views and viewing,” *Jewish History* 24, no. 2 (2010): 127–53, here 128–9, 140.

60. Maté Rigó, “Ordinary women and men: Superintendents and Jews in the Budapest yellow-star houses in 1944–1945,” *Urban History* 40, no. 2 (2013): 71–91, here 82.

61. H V-n, “De rättrogna,” *Dagens Nyheter*, March 5, 1905, 2–3.

62. Korbel, “Spaces of Gendered Jewish and Non-Jewish Encounters,” 95.

63. Paul Gilroy, *After Empire. Melancholia or Convivial Culture* (London: Routledge, 2004), xi, 155–57.

64. Magdalena Nowicka, and Steven Vertovec, “Comparing convivialities: Dreams and realities of living-with-difference,” *European Journal of Cultural Studies 17*, no. 4 (2014): 34–56.

65. Tim Corbett, Klaus Hödl, Caroline Kita, Susanne Korbel, and Dirk Rupnow, “Migration, Integration, and Assimilation: Reassessing Key Concepts in (Jewish) Austrian History,” *Journal of Austrian Studies* 54, no. 1 (2021): 1–28, here 10–18.

66. The concept of Jewish ‘difference’ was coined by Klaus Hödl and Lisa Silverman: Klaus Hödl, “From Acculturation to Interaction: A New Perspective on the History of the Jews in Fin-de-Siècle Vienna,” *Shofar* 25, no. 2 (2007): 82–103; Lisa Silverman, *Becoming Austrians: Jews and Culture between the World Wars* (Oxford: Oxford University Press, 2012), 6–10.

67. Korbel, “Spaces of Gendered Jewish and Non-Jewish Encounters.”

68. Vilma Neuwirth, *Glockengasse 29* (Vienna: Milena, 2008), 24.

69. Ibid., 23.

70. Corbett et. al., “Migration, Interaction, and Assimilation,” 10.

71. On the duality of popular and religious culture in Vienna’s Leopoldstadt, see Korbel, *Auf die Tour*, 62–7.

72. Korbel, “Spaces of Gendered Jewish and Non-Jewish Encounters,” 89.

73. Podcast *Bowery Boy*, Episode 246, December 17, 2017. Shared spaces occur at around 8:40.

74. Tilman Heil, “Are neighbours alike? Practices of conviviality in Catalonia and Casamance,” E*uropean Journal of Cultural Studies* 17, no. 4 (2014): 452–70.

75. Maja Hultman, “Atmospheres of the other: Building and feeling Stockholm’s orthodox synagogue,” *Emotion, Space & Society* 44 (2022): 6.

76. Neuwirth, *Glockengasse*, 24.

77. Ibid., 48.

78. Albert Lichtblau, “Antisemitismus 1900–1938: Phasen, Wahrnehmungen und Akkulturationseffekte,” in *Wien und die jüdische Erfahrung 1900–1938: Akkulturation – Antisemitismus – Zionismus*, eds. Barbara Eichinger, and Frank Stern (Vienna: Böhlau, 2009), 39–58.

79. See, for example: Bodenhamer et. al., eds., *Deep Maps and Spatial Narratives*.

